# White-matter free-water diffusion MRI in schizophrenia: a systematic review and meta-analysis

**DOI:** 10.1038/s41386-022-01272-x

**Published:** 2022-01-15

**Authors:** Inês Carreira Figueiredo, Faith Borgan, Ofer Pasternak, Federico E. Turkheimer, Oliver D. Howes

**Affiliations:** 1 Psychosis Studies Department, Institute of Psychiatry, Psychology and Neuroscience, King’s College London, London, UK; 2 Departments of Psychiatry & Radiology, Brigham and Women’s Hospital, Harvard Medical School, Boston, MA, United States of America; 3 Department of Neuroimaging, Institute of Psychiatry, Psychology & Neuroscience, Kings College London, London, UK; 4 Institute of Clinical Sciences (ICS), Faculty of Medicine, Imperial College London, Du Cane Road, London, UK; 5 H. Lundbeck UK, Valby, Denmark

## Abstract

White matter abnormalities, including increases in extra-cellular free-water, are implicated in the pathophysiology of schizophrenia. Recent advances in diffusion magnetic resonance imaging (MRI) enable free-water levels to be indexed. However, the brain levels in patients with schizophrenia have not yet been systematically investigated. We aimed to meta-analyse white-matter free-water levels in patients with schizophrenia compared to healthy volunteers.

We performed a literature search in EMBASE, MEDLINE, and PsycINFO databases. Diffusion MRI studies reporting free-water in patients with schizophrenia compared to healthy controls were included. We investigated the effect of demographic variables, illness duration, chlorpromazine equivalents of antipsychotic medication, type of scanner, and clinical symptoms severity on free-water measures.

Ten studies, including five of first episode of psychosis have investigated free-water levels in schizophrenia, with significantly higher levels reported in whole brain and specific brain regions (including corona radiata, internal capsule, superior and inferior longitudinal fasciculus, cingulum bundle, and corpus callosum). Six studies, including a total of 614 participants met the inclusion criteria for quantitative analysis. Whole-brain free-water levels were significantly higher in patients relative to healthy volunteers (Hedge’s g = 0.38, 95%, confidence interval (CI) 0.07 to 0.69, p = 0.02). Sex moderated this effect, such that smaller effects were seen in samples with more females (z = -2.54, p < 0.05), but antipsychotic dose, illness duration and symptom severity did not.

Patients with schizophrenia have increased free-water compared to healthy volunteers. Future studies are necessary to determine the pathological sources of increased free-water, and its relationship with illness duration and severity.

## Introduction

Schizophrenia is characterised by psychotic, negative and cognitive symptoms and is a leading cause of global disability[1]. Although its pathophysiology is still unknown, evidence indicates the involvement of disrupted early neurodevelopment, and aberrant function of cortical microcircuits, leading to a widespread network dysfunction and a cortical excitatory-inhibitory imbalance[1]. Current treatments target the dopaminergic system and are largely ineffective for the negative and cognitive symptoms of schizophrenia, the main contributors to the burden and morbidity of this disorder[2]. This highlights the need for greater understanding of the pathophysiology of the disorder to identify new treatment targets[3][4].

Multiple studies have found brain structural abnormalities in schizophrenia[5–8]. These have included studies using diffusion Magnetic Resonance Imaging (dMRI) to measure white matter microstructure by characterizing the amplitude and anisotropy of water diffusion, based on the principle that water tends to diffuse more freely along the longitudinal axis of axons than along their perpendicular axis[9]. Diffusion tensor imaging (DTI) provides unique information about white-matter microstructural properties that are useful in characterising pathophysiology in brain disorders[10]. Changes in fractional anisotropy (FA) and mean diffusivity (MD) are indicative of white matter pathology[11]. Studies using dMRI have shown reduced FA and altered MD in individuals with schizophrenia relative to controls [12–14].

However, one key limitation of DTI is that alterations in fast diffusing extraceullular water, such as free-water, can bias the estimation of DTI indices, confounding the interpretation of the signal changes. Increases in extracellular water can occur due to processes such as atrophy, changes in the extracellular matrix, and also due to inflammation[15–18] which, given the hypothesised role of neuroinflammation in schizophrenia[19–21], is of particular interest in the disorder. Thus, it is important to determine if there are alterations in extracellular water in schizophrenia.

Extracellular water can be quantified using free-water imaging[22]. This uses a bi-tensor model to characterise water diffusion as two compartments: one consisting of an unrestricted, isotropic compartment with a fixed diffusivity of free-water (FW), and another consisting of all remaining water molecules that are hindered or restricted by tissue membranes. From the tissue compartment, a measure of diffusion fractional anisotropy within the tissue (FA_T_) can be calculated. Because partial volume confounders from free-water-contaminated voxels (e.g., CSF) are eliminated[23], the FA_T_ measure is more specific to tissue alterations than FA measured using standard DTI [24–26].

There have been a number of recent studies to investigate free-water in schizophrenia. However, the magnitude of findings and the relationship to symptoms is not clear. To address this, we conducted a systematic review and meta-analysis to determine if there are significant FW alterations in schizophrenia, and to estimate the magnitude of any alterations, if relevant. In addition, we investigated methodological and clinical factors, including age, sex, duration of illness, treatment and symptom severity, that may influence findings. We hypothesized that patients with schizophrenia would have higher free-water values compared to healthy volunteers, and that illness severity would be positively associated with free-water values.

## Method

### Search strategy

In accordance with the PRISMA guidelines, EMBASE, MEDLINE, and PsycINFO databases were systematically searched from 2009 to 23 October 2020 using the following search terms: (1) “free-water” OR “free?water” AND (2) “diffusion tensor imaging” OR “diffusion magnetic resonance imaging” OR “dti” OR “diffusion mri” OR “neuroimaging” OR “imaging” AND (3) “schizophrenia” OR “psychosis” OR “psychotic”. The search criteria were registered on the international prospective register for systematic reviews (registration number CRD42020182173, review protocol accessible). The search and data extraction were independently checked by 2 different investigators (ICF and FB).

### Eligibility criteria

The inclusion criteria for the systematic review and meta-analysis were as follows: (1) original research article; (2) use of diffusion tensor imaging and the free-water bi-tensor model; (3) reports of free-water values, with sufficient information to determine the group mean and variance or effect size; (4) inclusion of schizophrenia, schizoaffective, or schizophreniform patients as determined by the Diagnostic and Statistical Manual of Mental Disorders (DSM); (5) inclusion of a control group of healthy volunteers with no history of psychiatric conditions.

Exclusion criteria for the systematic review were as follows: (1) studies that did not report original, peer-reviewed data, such as review articles and conference abstracts; (2) studies that did not report free-water imaging values; (3) studies not including patients with schizophrenia; (4) not including a healthy volunteer comparison group; (5) studies only reporting a free-water analysis in grey matter; (6) use of concurrent environmental manipulations (e.g. stress or food deprivation models).

A meta-analysis was conducted if there were at least 3 studies that used the same population, region of interest of interest in the brain, type of analysis (i.e. wholebrain analysis, brain regions of interest), and FW imaging method. All datasets included in the meta-analysis were independent.

### Data extraction

In accordance with the PRISMA guidelines, the following variables were extracted from all studies: (1) authors; (2) year of publication; (3) sample characteristics (sex (%female), age, diagnosis, duration of illness, medication status as reported chlorpromazine-equivalent estimates, duration of antipsychotic treatment, and sample size per group; (4) free-water imaging methods used, either multi or using a code adapted for single-shell; (5) regions of interest included (brain regions, white-matter tracts, and hemispheres); (6) type of analysis used (voxel-wise analysis, and tract-based spatial statistics (TBSS); (7) results (mean and variance of free-water values).

In cases where the mean and variance of free-water values were not reported, the mean and variance were extracted from tables or graphs using Web plot digitizer (WebPlotDigitizer 2018). In cases where it was unclear if datasets were independent, authors were contacted to confirm that this was the case. In cases where whole-brain analysis was separated into left versus right hemispheres, authors were contacted to provide whole-brain mean and standard deviation values. Studies where only a cluster of regions of interest were included in data analysis, were excluded from the analysis. When reported, we have converted the total BPRS score to an equivalent total PANSS score using the method described by Leucht et al, 2013[27].

## Statistical analysis

The main outcome measure in our analysis was the summary effect size (Hedge’s g) for the difference in FW values between patients with schizophrenia and healthy controls. All comparisons were conducted with the statistical programming language R Studio (version 3.3.2) using the “metafor” package. Standardized effect sizes (Hedges’ g using a 95% confidence interval and a significance level of p < 0.05 (two-tailed)) for individual studies were first estimated. An overall summary effect size was then calculated by entering these individual study effect sizes into a random effects meta-analytic model using restricted maximum likelihood estimation.

### Assesment of inconsistency and bias

Between-study inconsistency was estimated using the I^2^ value (I^2^ < 50% indicates low to moderate inconsistency, whereas I^2^ > 50% indicates moderate to high inconsistency). Publication bias was assessed in cases where there were at least 5 available studies by visual inspection of a funnel plot and the use of the Egger’s test. In cases where publication bias was suspected, a trim-fill analysis was conducted. If at least 5 studies were included in a meta-analysis, a leave-one-out sensitivity analysis was conducted to ensure that the results were not driven by a single study.

### Sensitivity analyses

Since previous literature has shown age, sex[28], and duration of illness-dependent effects on white-matter microstructure[12], meta-regressions were conducted to examine the effect of age, gender, duration of illness and symptom severity on FW values. We then compared studies including patients with first episode psychosis and chronic schizophrenia by fitting a meta-regression model where the subgroup category acted as the moderating variable of interest. If there was a statistically significant differences between subgroups, a separate random-effects meta-analysis was conducted for each sub-group. Meta-regressions were also used to investigate the effect of age (mean), gender (%male), type of scanner (1.5 vs. 3.0 Tesla), antipsychotic chlorpromazine-equivalent doses (mean), duration of illness (mean months) on free-water for the entire dataset. Given the evidence supporting significant free-water differences dependent on illness status[25], the meta-regressions were also used separately for patients with first episode psychosis (FEP) and chronic schizophrenia (CSZ). If at least 3 studies reported symptom severity using the same scale (e.g. Brief Psychiatry Rating Scale (BPRS), and/or Positive and Negative Symptoms Scale (PANSS), and/or Global Assessment of Functioning (GAF), we conducted a meta-regression to investigate the association between symptom severity and free-water for the entire sample, and separately for first episode of psychosis, chronic schizophrenia and healthy volunteers.

## Results

### Study sample and methodological characteristics

**Figure 1:**
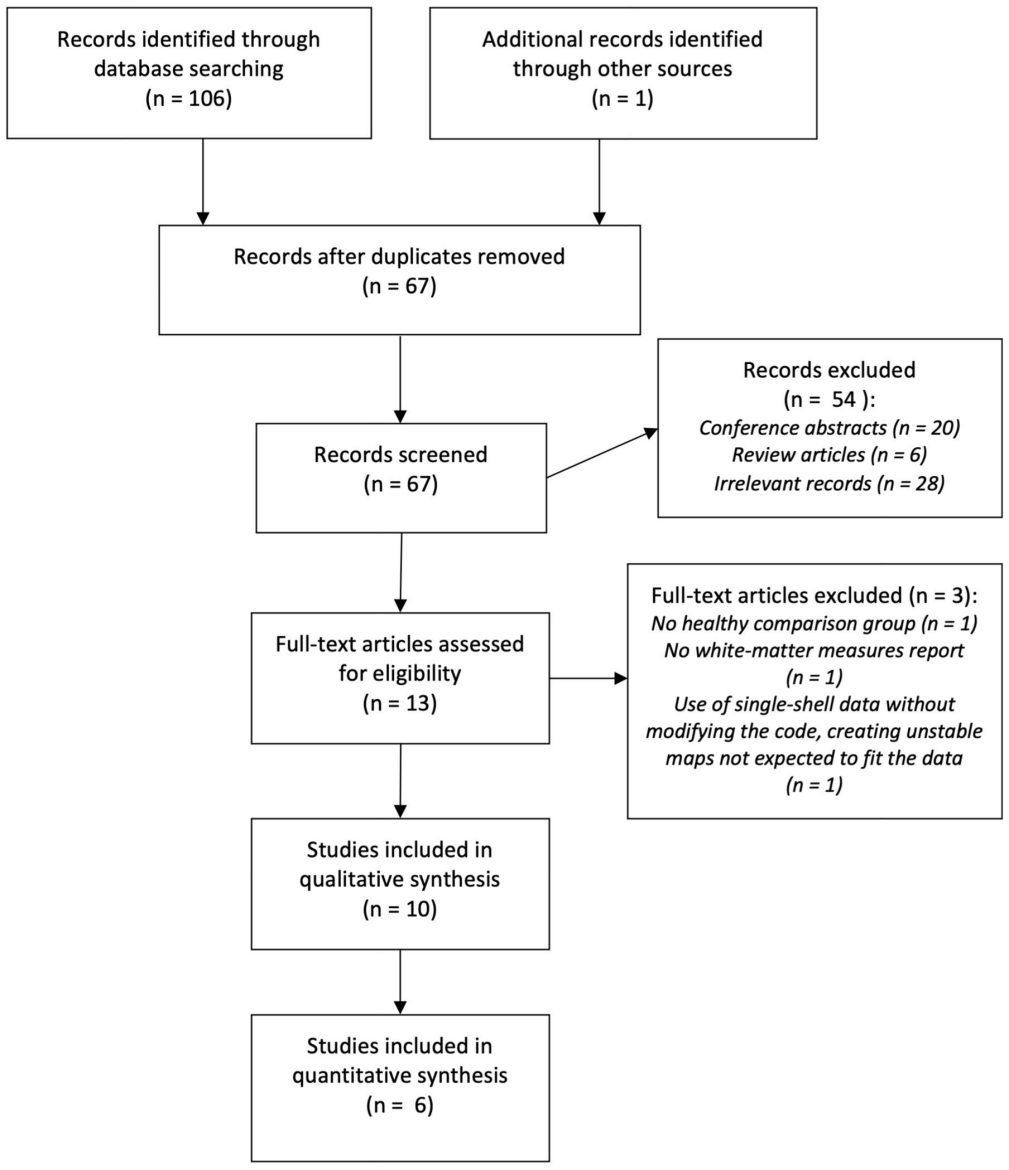
Study selection flow chart

The literature search identified 106 records. These were manually screened by two independent researchers (see Figure 1 for a study selection flow chart). In total, 10 articles were deemed eligible for inclusion in the systematic review, and 6 of these studies were suitable for inclusion in the meta-analysis.

The characteristics of the studies identified are summarised in Table 1. Of these, five studies were of patients in their first episode of illness, and five were of patients with chronic illnesses.

### Systematic review of studies investigating free-water in schizophrenia

The reported extracellular free-water differences between patients and healthy volunteers are summarised in Table 2. Of the ten studies, five studies[29–33] did not report a statistically significant difference between groups. This may be due to differences in duration of illness, given that patients included in those studies were diagnosed with chronic schizophrenia. The remaining five studies reported significantly higher free-water levels in schizophrenia.

Although there were insufficient data to investigate the association between free-water values and PANSS subscales, one study[25] reported a secondary analysis revealing patients with positive symptoms (olfactory hallucinations, thought disorder, pressured speech, and inappropriate affect) had significantly higher FW values relative to patients who were asymptomatic. One other study[32] also showed that total Scale for the Assessment of Positive Symptoms (SAPS) scores were significantly associated with increased free-water values in the right posterior thalamic radiatia and the left sagittal stratum.

Focusing on the studies that included patients with the diagnosis of first episode of psychosis, three studies[24][34][35] found differences in free-water levels between early onset patients and healthy volunteers. The only study that did not find a difference[33] used a scanner with a lower magnetic field (1.5T), which may have reduced the sensitivity to detect diffrerences.

### Meta-analysis of free-water

**Figure 2:**
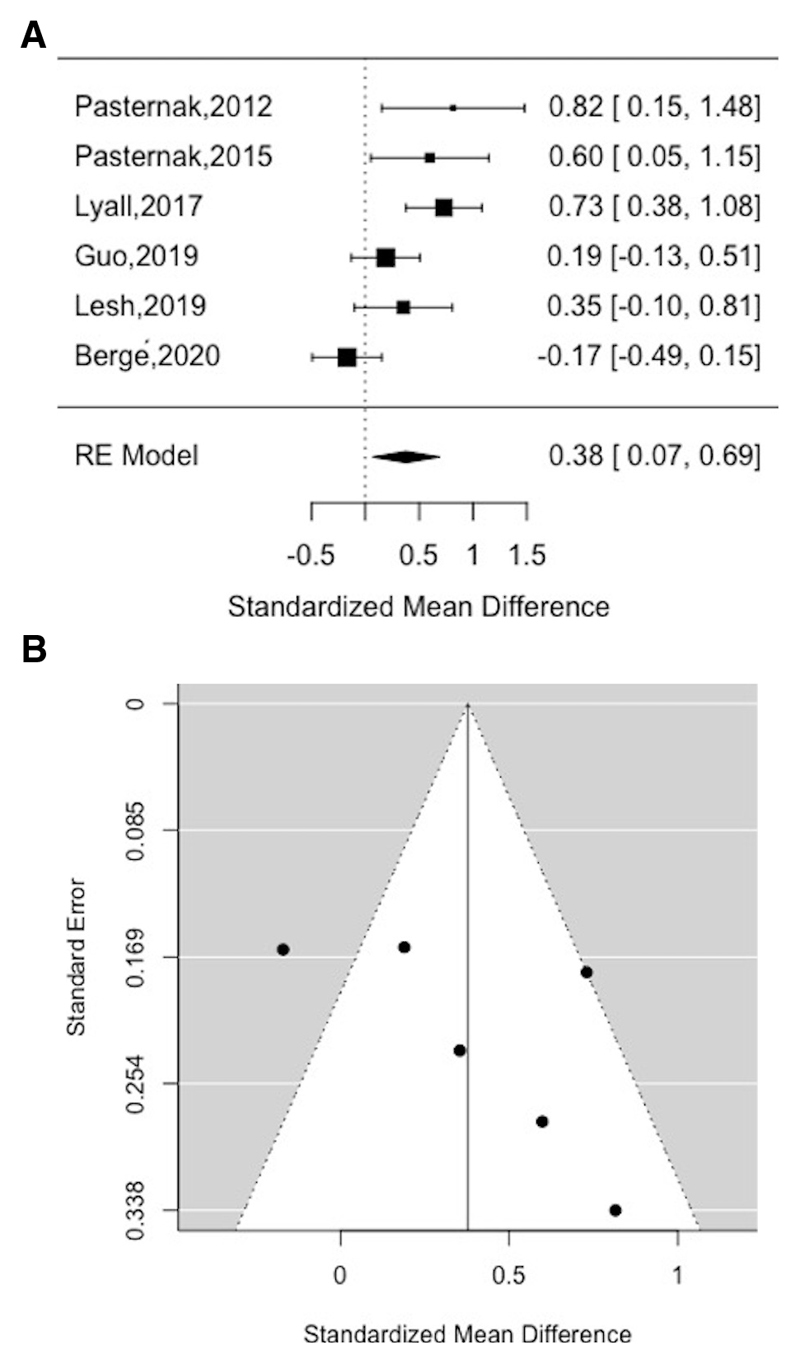
Standardized mean difference in FW in patients compared to healthy volunteers (A) and funnel plot for FW analysis (B)

The overall sample in the meta-analysis comprised 614 participants (288 patients with schizophrenia, 326 healthy volunteers). The mean age was 24 years (SD 8.7), and 234 (38.1%) of the participants were female. The average duration of illness was 36.7 months (SD 41.8) and mean chlorpromazine (CPZ) equivalent antipsychotic dose was 365.2 (SD 343) mg/day, although one study failed to report any medication equivalents[35]. Whole-brain free-water was significantly higher in patients relative to healthy volunteers. (Hedge’s g = 0.38, 95% confidence interval (CI) 0.07 to 0.69, p = 0.02) (see Fig. 2A, and Figure 2B for funnel plot). There were moderate to high levels of between-study inconsistency (I^2^ = 70.32, p < 0.01). However, Egger’s test indicated that there was no evidence of publication bias (z = 1.50, p > 0.05), and a trim-fill analysis indicated that there were no missing studies.

#### Effect of moderators

The magnitude of the effect size of free-water values between patients vs. controls did not significantly vary with age (z = 0.4653, p = 0.64), illness duration (z = 0.72, p = 0.47), type of scanner (z = -0.88, p = 0.38), CPZ equivalent antipsychotic doses (z = -0.13, p = 0.89), or total PANSS score (z = -0.11, p = 0.91). The magnitude of the effect size of free-water values between patients vs. controls significantly varied with sex (z = -2.54, p < 0.05), such that samples with a greater proportion of female patients associated with smaller free-water difference in patients relative to controls.

There were insufficient studies to investigate the effect of disease status (FEP vs CSZ) on free-water values.

## Discussion

Our main finding was that free-water levels in the white matter are higher in patients with schizophrenia compared to healthy volunteers. The magnitude of this effect did not vary with variables such as age, illness duration, total PANSS score, CPZ equivalent antipsychotic drugs, or type of scanner used for data acquisiton. However, a greater proportion of females resulted in smaller FW difference in patients relative to controls.

### Strengths and Limitations

Strengths include that this first study to perform meta-analyse of free-water measures in schizophrenia includes a moderately large sample size. However, we detected significant heterogeneity between studies. Our sensitivity analyses identified sex as a potential contributor to this.

In addition, five of the six studies included in the meta-analysis included individuals with the diagnosis of schizoaffective, or schizophreniform disorder, which may have contributed to the heterogeneity in the sample. There was also variability in the criteria used to match healthy controls with patients, which could contribute to heterogeneity. Nevertheless, all of the studies reported matching between patients and controls for age, gender and education.

Our findings are in accordance with previous studies highlighting that excessive free-water characterizes the white-matter pathology in early stages of schizophrenia, and other processes such as demyelination or axonal degeneration predominate with disease progression[16][25]. However, since only 1 out of 6 studies in our analysis included patients with the diagnosis of chronic schizophrenia, we were unable to further characterise the disease progression effect.

In regards to possible medication effects, we did not find a relationship with CPZ equivalent dose, suggesting antipsychotics are not substantially influencing our findings. However, since 4 studies did not report the number of unmedicated patients in the sample[16][29][34][36], we were unable to account for this in our meta-analysis. Therefore, differences in the use of medications may have contributed to high levels of heterogeneity across studies[37]. Notwithstanding these potential sources of variability, we used a random effects meta-analysis, which allows for heterogeneity in findings, although this approach underestimates the effect size relative to fixed effects approaches. Thus, the true effect may be larger than we report here. Another limitation was that there were too few studies to permit meta-analyses in specific regions. Thus, further studies reporting data on specific brain regions are needed to determine if there are regionally specific effects.

### Implications for understanding the pathophysiology of schizophrenia

Increased apparent diffusion coefficient (ADC), and MD levels[38] have consistently been reported in schizophrenia[10][39]. Our findings add to this, by indicating that there is increased extra-cellular free-water in white matter in people with schizophrenia as well. The fractional volume of the extracellular water, relative to the remaining water molecules diffusing in restricted conditions, is increased in pathological processes known to modify the interstitial extracellular space[40], such as vasogenic edema[23], and neuroinflammation[41]. Thus, the increased free-water in schizophrenia could be indicative of one or more of these processes, consistent with the hypothesis of immune activation in schizophrenia.

It has been hypothesised that the innate immune system triggers an inflammatory response in the brain in schizophrenia[42], in a process that also involves astrocytes and microglia [19][20], and leads to synaptic alterations and disruption of long-range connectivity, both of which have been reported in schizophrenia [43]. Other mechanisms could also contribute to the connection between schizophrenia and increased white-matter extracellular volume. In particular, a blood-brain barrier (BBB) disruption has been hypothesised to be consistent with the neuroimaging findings seen in the disorder[22][44], and in line with ideas that schizophrenia is a multi-system disorder[45]. When disrupted, abnormal trafficking of cells and molecules between the peripheral blood and the brain occur, allowing bone marrow-derived immune cells to cross into the nervous system[46], and increasing the extracellular fractional volume[47].

Further work is needed to determine if inflammatory states or other mechanisms underlie the increased free-water in schizophrenia. In particular, studies are needed in first episode patients, ideally combining free-water imaging with other neuroimaging methodologies such as PET measures of markers expressed on immune cells[20][48–50] and including measures of peripheral and central cytokines[19][51–53] in order to understand how these peripheral and central measures of inflammation are related. It is also important to recognise that extracellular changes in free-water could also reflect other biological factors such as decreased cell density[26]. However, altered cell density is not generally reported in schizophrenia[25].

We did not find that symptom severity moderated findings, indicating that our free-water findings are unrelated to symptoms. Neuroinflammation has been previously hypothesised as contributing to impaired neurocognitive functioning in schizophrenia[19][54]. Contrarily, a certain degree of acute inflammation is necessary to attain optimal function of the central nervous system[55], and even support healing in cases of brain injury[56]. Given the limited number of studies included in our meta-analysis that reported symptom ratings, this finding warrants further investigation before firm conclusions are drawn.

We did not find a relationship with CPZ equivalent dose, suggesting antipsychotics are not substantially influencing our findings. Critically, studies addressing the effect of antipsychotic treatment in microglia have shown conflicting results, with some *in vitro* studies showing a reduction in microglial activation[57], and *in vivo* studies reporting an increase with olanzapine[58], but a reduction with risperidone[59]. Thus, further work is needed to comprehend if antipsychotic treatment could have affected our results.

We found that patients with schizophrenia show greater white-matter free-water levels relative to healthy volunteers with a moderate effect size. There was significant heterogeneity, which could be partly due to differences in sex between studies *given our finding that studies with more females showed greater elevations in free water in patients relative to controls*. This finding is consistent with previous free-water results showing that female FEP patients exhibit significant increases in free-water values compared to female healthy volunteers[35]. However, there is not a consensual explanation for these findings, and future work examining sex differences in these neuroinflammatory parameters is warranted.

We did not investigate differences in FAT in our meta-analysis. Future studies are needed to understand the magnitude of findings in schizophrenia and implications of this parameter.

These findings extend the existing understanding of the extracellular and white matter changes in schizophrenia and they highlight the possible role of extracellular pathologies such as neuroinflammation in the pathophysiology of the disorder.

## Figures and Tables

**Table 1:** Studies examining extracelluar free-water differences between patients with schizophrenia and healthy volunteers^a^

Source	Setting	No. of patients	Patient Age, Mean (SD), y	Patient Gender, % Male	*DSM* Diagnoses	Disease status (FEP, CSZ)	Duration of illness (months), Mean (SD)	PANSS total score Mean (SD)	No. of controls	CPZ equivalent, Mean (SD)	Type of scanner	Neuroimaging ROIs
Pasternak et al, 2012	United States of America (USA)	18	21.6 (4.3)	77.8	Schizophrenia, schizoaffective, or schizophreniform	FEP	6.2 (4.3)	79(20)	20	472.2 (651.3)	3 T	Whole-brain
Berge et al, 2020	Spain, The Nederlands	59	22.1 (5.9)	64.4	Schizophrenia, schizoaffective, or schizophreniform	FEP	4.6 (3.3)	82.3 (27.6)	101	527.5 (409.4)	3 T, 1.5 T	Whole-brain
Lesh et al, 2019	USA	36	21.4 (3.4)	69.4	Schizophrenia, schizoaffective, or schizophreniform	FEP	Not reported	84 (19)	40	226.6 (168.7)	3 T	Whole-brain
Lyall et al, 2017	USA	63	21.4 (4.9)	73	Schizophrenia, schizoaffective, or schizophreniform	FEP	Not reported	79 (14)	70	Not reported	3 T	Whole-brain
Mandl et al, 2015	The Nederlands/ USA	40	26.8 (5.8)	92.5	Schizophrenia	CSZ	25.1 (17.4)	62.3 (15.8)	40	Not reported	1.5 T	Bilateral uncinate, bilateral arcuate fasciculus, bilateral inferior longitudinal fasciculus, bilateral inferior fronto-occipital fasciculus, genu of the corpus callosum, splenium of the corpus callosum, bilateral cingulum bundle
Oestreich et al, 2017	Australia/ USA/ Ireland	281	39 (11.2)	73	Schizophrenia	CSZ	15.1 (9.8)	Not reported	188	Not reported	1.5 T	42 ENIGMA-DTI^b^ ROIs, of which 38 were bilateral (anterior corona radiata, anterior limb of internal capsule, cingulum (cingulate gyrus, (hippocampus), corona radiata, corticospinal tract, external capsule, fornix/stria terminalis, internal capsule (IC), inferior occipito-frontal fasciculus, posterior corona radiata, posterior limb of IC, posterior thalamic radiation, retrolenticular part of IC, superior corona radiata)
Oestreich et al, 2016	Australia/ Ireland	86	39.5 (9.9)	77.8	Schizophrenia	CSZ	180.4 (103)	Not reported	28	Not reported	1.5 T	Cingulum bundle, uncinate fasciculus and fornix, internal capsule
Pasternak et al, 2015	USA	29	46.6 (9.5)	86.2	Schizophrenia	CSZ	15 (10.5)	86.9(28.7)	25	451 (273)	3 T	Whole-brain
Gurholt et al, 2020	Norway/ Sweden/ USA	30	51.1 (7.9)	73.7	Schizophrenia, schizoaffective	CSZ	27.6 (8)	Not reported	42	409.8 (325.2)	3 T	ENIGMA-DTI^b^ ROIs (anterior corona radiata, anterior limb of internal capsule, body of corpus callosum, corpus callosum, cingulum, cingulum (hippocampal portion), corona radiata, corticospinal tract, external capsule, fornix, fornix stria terminalis, genu of corpus callosum, IC, inferior fronto occipital fasciculus, posterior corona radiata, posterior limb of IC, posterior thalamic radiation, retrolenticular part of IC, splenium of corpus callosum, superior corona radiata, superior fronto-occipital fasciculus, superior longitudinal fasciculus, sagittal stratum, uncinate)
Guo et al, 2019	USA	83	21.0 (3.2)	80.7	Schizophrenia	FEP	6.6 (5.8)	Not reported	70	240.6 (283)	1.5 T	Whole-brain

Abbreviations: *SD* standard deviation, *CPZ* chlorpromazine, *ROIs* regions of interest, *FEP* first episode of psychosis, *CSZ* chronic schizophrenia, *PANSS* Positive and Negative Symptoms Scale, *T* Tesla

a only Berge et al and Guo et al used longitudinal designs

b http://enigma.ini.usc.edu/protocols/dti-protocols/

**Table 2:** Summary of extracellular free-water differences between patients with schizophrenia and healthy volunteers. ↑=significantly higher in schizophrenia, ↓=significantly lower in schizophrenia.

Source	Neuroimaging ROIs	Included in meta-analysis (Yes/No)	FW levels in schizophrenia relative to controls
Pasternak et al, 2012[24]	Left hemisphere *versus* right hemisphere*	Yes	↑
Bergé et al, 2020[34]	Superior Corona Radiata, Internal Capsule, Superior Longitudinal Fasciculus, Inferior Longitudinal Fasciculus, and body of Corpus Callosum and Thalamic Radiation*	Yes	↑
Lesh et al, 2019[29]	Lateral frontal cortex, right rostral anterior cingulate, bilateral temporal cortex extending into the insula, left hemisphere inferior parietal cortex, left hemisphere posterior cingulate, and occipital cortex (vertex analysis)	Yes	No significant differences
Lyall et al, 2017[35]	Whole-brain white matter*	Yes	↑
Mandl et al, 2015[30]	Left uncinate fasciculus, the right inferior longitudinal fasciculus	No (no whole-brain analysis)	No significant differences
Oestreich et al, 2017[31]	interhemisphere tracts	No (data extraction not possible)	No significant differences
Oestreich et al, 2016[36]	cingulum bundle, uncinate fasciculus and fornix, internal capsule*	No (no whole-brain analysis)	↑
Pasternak et al, 2015[25]	Left hemisphere (anterior, superior and posterior corona radiata, parts of the genu and splenium of the corpus callosum)*	Yes	↑
Gurholt et al, 2020[32]	anterior corona radiata, anterior limb of internal capsule, body of corpus callosum, corpus callosum, cingulum, cingulum (hippocampal portion), corona radiata, corticospinal tract, external capsule, fornix, fornix stria terminalis, genu of corpus callosum, internal capsule, inferior fronto occipital fasciculus, posterior corona radiata, posterior limb of internal capsule, posterior thalamic radiation, retrolenticular part of IC, splenium of corpus callosum, superior corona radiata, superior fronto-occipital fasciculus, superior longitudinal fasciculus, sagittal stratum, uncinate	No (no wholebrain analysis)	No significant differences
Guo et al, 2019[33]	whole-brain white matter	Yes	No significant differences

* All ROIs with statistically significant increase in FW
